# Knockout of the gamma subunit of the AP-1 adaptor complex in the human parasite *Trypanosoma cruzi* impairs infectivity and differentiation and prevents the maturation and targeting of the major protease cruzipain

**DOI:** 10.1371/journal.pone.0179615

**Published:** 2017-07-31

**Authors:** Claudia Maria do Nascimento Moreira, Cassiano Martin Batista, Jessica Chimenes Fernandes, Rafael Luis Kessler, Maurilio José Soares, Stenio Perdigão Fragoso

**Affiliations:** 1 Laboratory of Molecular Biology of Trypanosomatids, Instituto Carlos Chagas/Fiocruz, Curitiba - PR, Brazil; 2 Laboratory of Cell Biology, Instituto Carlos Chagas/Fiocruz, Curitiba - PR, Brazil; 3 Laboratory of Functional Genomics. Instituto Carlos Chagas/Fiocruz, Curitiba - PR, Brazil; University of Georgia, UNITED STATES

## Abstract

The AP-1 Adaptor Complex assists clathrin-coated vesicle assembly in the *trans*-Golgi network (TGN) of eukaryotic cells. However, the role of AP-1 in the protozoan *Trypanosoma cruzi*—the Chagas disease parasite—has not been addressed. Here, we studied the function and localization of AP-1 in different *T*. *cruzi* life cycle forms, by generating a gene knockout of the large AP-1 subunit gamma adaptin (TcAP1-γ), and raising a monoclonal antibody against TcAP1-γ. Co-localization with a Golgi marker and with the clathrin light chain showed that TcAP1-γ is located in the Golgi, and it may interact with clathrin *in vivo*, at the TGN. Epimastigote (insect form) parasites lacking TcAP1-γ (TcγKO) have reduced proliferation and differentiation into infective metacyclic trypomastigotes (compared with wild-type parasites). TcγKO parasites have also displayed significantly reduced infectivity towards mammalian cells. Importantly, TcAP1-γ knockout impaired maturation and transport to lysosome-related organelles (reservosomes) of a key cargo—the major cysteine protease cruzipain, which is important for parasite nutrition, differentiation and infection. In conclusion, the defective processing and transport of cruzipain upon AP-1 ablation may underlie the phenotype of TcγKO parasites.

## Introduction

The protozoan *Trypanosoma cruzi* (Euglenozoa: Kinetoplastea) is the causative agent of Chagas disease, which affects an estimated 6 to 7 million people worldwide, mostly in Latin America (WHO, 2016, http://www.who.int/mediacentre/factsheets/fs340/en/). During its life cycle this parasite alternates between an invertebrate host (hematophagous triatomine insects) and a mammalian host, with the four following well characterized developmental stages: bloodstream trypomastigotes and intracellular amastigotes, which are observed in the vertebrate hosts, and epimastigotes and metacyclic trypomastigotes, which are found in the insect digestive tract [1].

All *T*. *cruzi* developmental stages have lysosome-related organelles (LROs) [2]; however, in the epimastigote form, LROs have the additional and unique ability of storing cargo and they are called “reservosomes” [3]. Reservosomes are located at the posterior region of the parasite and accumulate proteins and lipids ingested from the extracellular medium by endocytosis, which occurs at the anterior region of the parasite. They also contain several hydrolases that are acquired from the secretory pathway and crucial for the digestion of the endocytosed material [4,5]. Reservosomes disappear during the differentiation of epimastigotes into metacyclic trypomastigotes (metacyclogenesis) [6,7], a process that can be triggered by nutritional stress [8]. It is possible that the content of reservosomes is mobilized as energy source during metacyclogenesis. Nutritional stress would trigger reservosome acidification, activating its hydrolases and altering organelle activity from a ‘pre’ to a ‘full’ lysosomal state. The consumption of stored material triggered by nutritional stress would explain reservosome disappearance during metacyclogenesis [7]. However, a recent study suggested that LROs from metacyclic trypomastigotes may arise directly from reservosomes [9].

Cruzipain, the major cysteine protease of *T*. *cruzi*, is highly expressed in epimastigotes [10] and accumulates in reservosomes [3] along with its inhibitor, chagasin [11]. Cruzipain plays an important role in parasite nutrition, differentiation and infectivity [12–16], and it is synthesized and processed in the endoplasmic reticulum/Golgi complex. However, it is not yet clear how cruzipain is targeted to reservosomes [17]. Newly formed reservosomes originate at the anterior region of the parasite, close to the Golgi complex [14,18], and cruzipain was detected in vesicles budding from the *trans*-Golgi network (TGN) that seemed to fuse with newly formed reservosomes [18]. These data indicate that the secretory pathway—which has the TGN as a key cargo-sorting hub—plays an important role in reservosome formation.

Cargo-containing vesicles bud from the TGN to different destinations by a regulated process that envolves the assembly of specific cytosolic cargo adaptors onto TGN membranes [19,20]. Cargo adaptors recognize an appropriate sorting signal in the cytosolic domain of cargo molecules and pack them, aided by auxiliary proteins, into vesicles that are delivered to their target sites at the endocytic or secretory pathways [21]. Eukaryotic cells have different cargo adaptors, including cytosolic proteins from the family of heterotetrameric adaptor complexes (APs) [22]. All five AP complexes identified to date (AP-1 to AP-5) consist of two large subunits of ~100 kDa (β1- β5, and either α, γ, δ, ε or ζ), a medium subunit of ~50 kDa (μ1–μ5) and one small subunit of ~20 kDa (σ1–σ5) [22,23].

The AP-1, AP3 and AP-4 complexes are involved in protein sorting at the TGN [19,20], while only AP-1 and AP-2 clearly recruit clathrin [24].

The *T*. *cruzi* genome contain the full set of genes for AP-1 to AP-4 complexes, but AP-5 appears to be absent in trypanosomatids [25,26]. The related parasites *T*. *brucei* and *Leishmania* spp. are only capable of assembling three of the AP adaptor complexes: *T*. *brucei* lacks AP-2, while AP-4 is absent in *Leishmania*. AP-1 is responsible for the transport of lysosomal hydrolases from the trans-Golgi Network (TGN) to endosomes in eukaryotic cells [19,27], a function that is conserved in primitive eukaryotes [28,29]. AP-1 appears to be ubiquitous in trypanosomatids [25] and studies indicate that it plays important roles in endosomal trafficking in *T*. *brucei* and *Leishmania mexicana*. Knockdown of the μ1 and γ subunits of the *T*. *brucei* AP-1 complex by RNA interference was lethal to both procyclic (insect form) and bloodstream forms of the parasite in axenic cultures [30,31]. In contrast, knockout mutants for the μ1 or σ1 subunits of the AP-1 complex in *L*. *mexicana* were viable in culture, but they were unable to infect macrophages or mice [32]. Although viable, *Leishmania* AP-1 mutants displayed defects in lysosome and lipid transport as well as in flagellar biogenesis [33]. Both μ1 and σ1 adaptins of *L*. *mexicana* AP-1 are localized to endosomes and TGN vesicles [33]. Overall, these data indicate that the AP-1 complex plays important roles in endosomal trafficking in trypanosomatids. Nevertheless, the role of APs in the endocytic and secretory pathways in *T*. *cruzi* remains unexplored.

Here, we investigated the function of AP-1 in *T*. *cruzi*, taking advantage of the highly polarized endocytic and secretory pathways in the epimastigote stage, where secretion and exocytosis/endocytosis sites (the Golgi complex and flagellar pocket/cytostome, respectively) are found at the anterior while the final compartments of the endocytic pathway (the reservosomes) are in the posterior. We show here that gene knockout of the *T*. *cruzi* AP-1 gamma subunit TcAP1-γ blocks the transport of cruzipain to the reservosomes and impairs parasite proliferation, metacyclogenesis and infectivity *in vitro*.

## Materials and methods

### Parasite

Epimastigote forms of *T*. *cruzi* clone Dm28c [8] were kept at 28°C in liver infusion tryptose (LIT) medium supplemented with 10% inactivated fetal bovine serum (FBS), with weekly passages.

Metacyclic trypomastigotes were obtained from the differentiation of epimastigotes in the chemically defined medium TAU3AAG, as previously described [34,35]. Epimastigotes in late exponential growth phase (5–6 x 10^7^ cells ml^-1^) were harvested from LIT medium by centrifugation and subjected to nutritional stress by incubation for 2 h (at 28°C) in triatomine artificial urine (TAU) medium (190 mM NaCl, 17 mM KCl, 2 mM MgCl_2_, 2 mM CaCl_2_, and 8 mM sodium phosphate buffer, pH 6.0) at a density of 5 x 10^8^ cells ml^-1^. The cell suspension was then inoculated (to a density of 5 x 10^6^ cells ml^-1^) in culture flasks containing TAU3AAG medium (TAU medium supplemented with 50 mM sodium glutamate, 10 mM L-proline, 2 mM sodium aspartate and 10 mM glucose) pre-warmed at 28°C, and incubated for 72h. After incubation, the metacyclic trypomastigotes found in the culture supernatant were purified by DEAE cellulose chromatography, as previously described [36].

Intracellular amastigotes were obtained from the differentiation of metacyclic trypomastigotes in VERO cells (ATCC CCL-81). VERO cells were grown in Dulbecco’s Modified Eagle’s medium (DMEM; Sigma) supplemented with 5% FBS and cultivated (at 37°C, in a humidified atmosphere with 5% CO_2_) until they reached 50–70% confluence. Cell monolayers were then infected with metacyclic trypomastigotes (150 parasites/host cell) obtained by metacyclogenesis as described above. After 24 h the medium was discarded (to remove non-adherent parasites), cultures were washed once with PBS and new medium was added to culture flasks. Amastigotes were obtained by disrupting VERO cells 10 days after infection and harvesting parasites by centrifugation at 1,000 x *g* for 5 min.

### Growth Curve and Metacyclogenesis quantification

For growth curve analysis, epimastigote cultures were established at a density of 1 x 10^6^ cells ml^-1^ and population growth was monitored for seven days, with cell counting in a Z series Coulter Counter (Beckman Coulter, CA, USA). Experiments were performed in technical and biological triplicates and the data were analyzed with the t-test using GraphPad software.

To quantify metacyclogenesis, epimastigotes were allowed to differentiate *in vitro* as described above (see “Parasite”), and the number of metacyclic trypomastigotes was counted in a Neubauer chamber after 72h of incubation in TAU3AAG medium (which corresponds to the peak of differentiation into metacyclic trypomastigotes). Experiments were performed in technical triplicates.

### Recombinant protein expression and purification

Nucleotides 1,609 to 2,091 (hinge region) of the gene encoding the *T*. *cruzi* AP-1 γ adaptin (TcAP1-γ, gene ID TcCLB.508257.260, from tritrypdb.org) were amplified by PCR using the primers HingeF and HingeR (Table 1), which contain the *attB*1 and *attB*2 recombination sites, respectively. These sites allowed the insertion of the PCR fragment into the pDONR 221 vector from the Gateway recombination cloning system (Thermo Scientific, MA, USA). The resulting recombinant plasmid (entry clone) was then used to transfer the hinge fragment DNA cassette (by recombination) into the expression vector pDEST17 from the Gateway platform. *Escherichia coli* strain BL21(DE3)STAR was used to produce the histidine-tagged TcAP1-γ hinge domain by induction with 1 mM IPTG. Inclusion bodies containing the insoluble recombinant protein were purified as previously described [37] and then solubilized in Laemmli sample buffer (60 mM Tris-HCl, pH 6.8, 10% glycerol, 2% SDS, 5% 2-mercaptoethanol and 0.01% bromophenol blue) at 100°C, for 10 min. The recombinant protein was recovered by electroelution from SDS-PAGE gels, as previously described [37].

**Table 1:** Primer sequences used in this work.

PRIMERS	SEQUENCES (5’- 3’)
Hinge F	**GGGGACAAGTTTGTACAAAAAAGCAGGCTTC**ATGAAAATCGCCTCCAGGTATCC
Hinge R	**GGGGACCACTTTGTACAAGAAAGCTGGGTCCTA**ACAATCAAACACACATGCACTTCAGG
UPS_KpnI	GTGGGTACCGTGTGTTGTGTTGTCCCTTTTTTTTTGTG
UPS_SalI	ACCGTCGACCGGCAACGATCAAGTCACGAAGTCTCGCA
DOWN_BamHI	GCGTGTGTGTGTGTGGATCCCTTTGTCGTCACGGAGAGAA
DOWN_XbaI	CGCCAGCACAATCTAGACATAGCTAGACTGAAACT
AP-1-γ_attB1	**GGGGACAAGTTTGTACAAAAAAGCAGGCTTC**ATGGAGGGGAAACTTGAGTCG
AP-1-γ_attB2	**GGGGACCACTTTGTACAAGAAAGCTGGGTCC**AACTCCTGCGTCACCTGA

*attB*1 and *attB*2 recombination sequences (for use with the Gateway cloning system) are shown in bold. Restriction sites are underlined.

### Monoclonal antibody (mAb) production

Four young adult male BALB/c mice (30–45 day-old) were inoculated five times with 20 μg of the histidine-tagged TcAP1-γ hinge domain at one-week intervals. The first four doses were administered by the intraperitoneal route using Alu-Gel-S (Serva, Heidelberg, Germany) as adjuvant. The fifth dose of antigen was inoculated by the intravenous route with no adjuvant. Three days after the last immunization, the animals were anesthetized with ketamine/xylazine solution (100 and 10 mg kg^-1^, respectively) by the intraperitoneal route, bled by cardiac puncture to obtain post-immune sera and euthanized by cervical dislocation. The spleens were aseptically removed to obtain antibody-secreting cells, which were fused with Ag8XP3653 myeloma cells to produce hybridomas, as previously described [38]. Positive hybridomas were selected by indirect ELISA, Western blot, and indirect immunofluorescence, and stable hybridomas were cloned by limiting dilution. The Mouse Rapid ELISA mAb Isotyping Kit (Thermo Scientific) was used to identify immunoglobulin isotypes. A positive monoclonal antibody against TcAP1-γ—named 211.F7—was selected using the screening procedure described above and was used throughout this work.

### Ethics statements

Experiments with animals were approved by the Ethics Committee for Animal Research of Fundação Oswaldo Cruz (Oswaldo Cruz Foundation, FIOCRUZ, attached to the Brazilian Ministry of Health, Rio de Janeiro, Brazil) under protocol number LW-15/13, which follows the guidelines on animal care of the Brazilian Council for the Control of Animal Experimentation (CONCEA).

### Western blot

Epimastigotes, *in vitro*-derived metacyclic trypomastigotes and isolated intracellular amastigotes obtained as described above (see “Parasites”) were washed in PBS, resuspended in PBS containing a protease inhibitor cocktail (Roche, Basel, Switzerland), and lysed by the addition of 4x Laemmli sample buffer, which was followed by heating at 100°C for 5 min. Lysates containing an equivalent of 1 x 10^6^ cells μl^-1^ were fractionated by SDS-PAGE and transferred onto nitrocellulose membranes (Hybond C, GE Healthcare, PA, USA) according to standard protocols [39]. Membranes were blocked with 5% non-fat dry milk and 0.05% Tween 20 in PBS (blocking solution) then, they were probed with one of the following primary antibodies (diluted in blocking solution): the monoclonal antibody 211.F7, against the Tcγ hinge domain (1:300); anti-cruzipain (1:1500) antiserum that recognizes both the zymogen and the mature form of cruzipain [40]; or a mouse anti-TcGAPDH antiserum (1:8,000) [41], as a loading control. Membranes were then incubated with horseradish peroxidase-conjugated anti-mouse IgG antibodies (Thermo Scientific product #31430) and diluted to 1:7,500 in blocking solution. The SuperSignal West Pico Chemiluminescent System (Thermo Scientific) was used for antibody binding detection according to the manufacturer’s instructions.

### Immunofluorescence microscopy

Parasites were washed twice in PBS, fixed for 10 min at room temperature with 4% paraformaldehyde, washed twice in PBS and then adhered for 20 min to 0.1% poly-L-lysine-coated coverslips. Then, cells were permeabilized for 5 min with 0.5% Triton X-100 in PBS, washed with PBS and blocked with 1.5% bovine serum albumin in PBS for 1 h. For cell surface labeling of WT and γKO amastigotes and metacyclic trypomastigotes, the parasites were fixed with paraformaldehyde without Triton X-100 treatment. Samples were then incubated for 1 hour at 37°C with either the anti-TcAP1-γ mAb 211.F7 (1:80) or anti-cruzipain antiserum (1:500) diluted in PBS (pH 7.4) containing 1.5% BSA (blocking buffer). Coverslips were washed three times with PBS and then incubated with goat anti-mouse IgG secondary antibodies coupled to Alexa Fluor 488 or Alexa Fluor 594 (Thermo Scientific; both at 1:600) in blocking buffer. Finally, samples were washed three times in PBS, incubated for 5 min with Hoechst 33342 (1 μg ml^-1^; Thermo Scientific), mounted with Prolong Gold (Thermo Scientific) and examined in a Leica SP5 confocal laser microscope (Leica Microsystems, Wetzlar, Germany).

For co-localization with TcAP1-γ, transfectant epimastigotes expressing the tagged Golgi marker Huntingtin interacting protein TcHIP/AC [42] or the tagged clathrin light chain TcCLC/AC [43] (where AC stands for the protein C/protein A ‘PTP’ epitope tag; [44]) were fixed, adhered and permeabilized as described above. Then, they were incubated for one hour at 37°C with the anti-TcAP1-γ mAb 211.F7 (1:80) and with a rabbit IgG anti-protein A antibody (1:40,000) (Sigma-Aldrich, MO, USA). Samples were washed three times in PBS, incubated with a goat anti-mouse antibody coupled to Alexa Fluor 488 (1:600) and a goat anti-rabbit antibody coupled to Alexa Fluor 546 (1:600), and processed for fluorescence microscopy as described above.

### Transmission electron microscopy

Epimastigotes in exponential growth phase (2–3 x 10^7^ cells ml^-1^) were collected by centrifugation and washed in PBS. The cells were then fixed with 2.5% glutaraldehyde in 0.1 M phosphate buffer (pH 7.2). After that, cells were washed once with 0.1 M phosphate buffer, once with 0.1 M cacodylate buffer and post-fixed with 1% osmium tetroxide/0.8% potassium ferricyanide/5 mM CaCl_2_ in 0.1 M cacodylate buffer (pH 7.2). Cells were dehydrated in graded acetone, infiltrated in acetone/PolyBed 812 mixture and embedded in PolyBed 812 resin (PolySciences, Warrington, PA, USA). Ultrathin sections were stained with uranyl acetate and lead citrate before examination in a JEOL JEM-1400 Plus transmission electron microscope at 80kV.

### Gene knockout

Flanking sequences of the *TcAP1-γ* gene were amplified from *T*. *cruzi* Dm28c genomic DNA by PCR using the primer pairs UPS_KpnI and UPS_SalI (5’ flank, 658 bp) and DOWN_BamHI and DOWN_XbaI (3’ flank, 652 bp) (Table 1). The 5’ flank fragment was inserted into the KpnI and SalI sites of pTc2KO-neo and pTc2KO-hyg, which carry the neomycin and hygromycin B resistance genes, respectively [45]. The 3’ flank fragment was then cloned into BamHI and XbaI of the recombinant plasmids containing 5’ flank fragments. The resulting plasmids were named pTc2KO-AP-1γ-neo and pTc2KO-AP-1γ-hyg (S1 Fig).

To produce the *TcAP1-γ* gene knockout, the targeting cassettes (5’flank-NEO-3’flank and 5’flank-HYG-3’flank) were amplified using UPS_KpnI and DOWN_XbaI primers and transfected into *T*. *cruzi* epimastigotes as previously described [37]. First, parasites were transfected with the 5’flank-NEO-3’flank cassette and incubated for 24 h in LIT medium before selection in LIT medium with 500 μg ml^−^1 G418. After 4 to 5 weekly passages in selection medium, G418-resistant transfectants were recovered, while no growth was observed in control cultures transfected without DNA. The G418-resistant parasite population was then transfected with the 5’flank-HYG-3’flank cassette, incubated for 24h in LIT medium, and selected in LIT medium supplemented with 500 μg ml^-1^ G418 and 500 μg ml^-1^ hygromycin B. Resistant transfectants were selected after 5 to 8 weekly passages. Individual clones from the double resistant parasite population were obtained by flow cytometry sorting. Briefly, epimastigote forms (1 x 10^6^ cells) were resuspended in 1 ml PBS. The parasites were analyzed by FACSAria II (BD) and *T*. *cruzi* autofluorescence helped to identify the cell population. The most homogeneous population was selected by forward scatter (FSc) vs. side scatter (SSc) contour plot and subjected to cell sorting. The single cell precision mode from FACSDiva (BD) software was used to directly sort single cells into 96-well plate containing 100 μl of LIT medium/well, that was supplemented with G418 (500 μg ml^-1^) and hygromycin B (500 μg ml^-1^). Parasites were kept at 28°C in a humidified atmosphere with 5% CO2. Parasite growth was observed 15–20 days from cell sorting. Clones were screened for the lack of TcAP1-γ expression by Western blot.

### Pulsed-field gel electrophoresis (PFGE) and Southern blot

To confirm TcAP1-γ knockout, chromosomes from wild-type (WT) and TcγKO parasites were fractionated by PFGE as previously described [46]. Chromosomes from *Hansenula wingei* (Biorad Laboratories, CA, USA) were used as molecular mass standards. After electrophoresis, gels were stained with 0.5 μg ml^−1^ ethidium bromide, imaged and processed by Southern blotting according to standard protocols [39]. After transfer to nylon membranes, specific DNA sequences were detected by hybridization with probes for the entire coding sequences of *TcAP1-γ*, *NEO* and *HYG* genes. Probes were radioactively labeled with [α-^32^P]-dCTP using the megaprime DNA labeling system (GE Healthcare).

### Complementation of TcAP1-γ KO parasites

For TcAP1-γ KO complementation, the *TcAP1-γ* gene was amplified by PCR (primers AP-1-γ_attB1 and AP-1-γ_attB2, Table 1), cloned (as described above) into pDONR 221 from the Gateway cloning system (entry clone), and then transferred to pTcGW-bsd, yielding pTcGW-AP-1γ-bsd (S2 Fig). The expression plasmid pTcGW-bsd is a modified version of the pTcGW 1.1 series Gateway expression vectors constructed for constitutive expression and selection in *T*. *cruzi* [47,48]. TcγKO parasites were transfected with 15 μg of pTcGW-AP-1γ-bsd and selected in LIT medium containing 15 μg ml^-1^ Blasticidin-S (Sigma-Aldrich). The expression and localization of TcAP1-γ in the complemented (add-back) cells were analyzed by Western blot and immunofluorescence microscopy, respectively, using the anti-TcAp1-γ mAb 211.F7. The TcAp1-γ-complemented parasites [TcγKO(pTc-AP1γ)] were further tested for their capacity to proliferate, differentiate and infect cells as well as for the restoration of cruzipain transport to the reservosomes.

### High-content quantification of *T*. *cruzi* infectivity

Two mammalian cell lines from distinct cell lineages were used for *in vitro* high-content analysis of parasite infectivity, HeLa cells (ATCC CCL-2; epithelial lineage) and VERO cells (ATCC CCL-81; fibroblast lineage). Cells were kept at 37°C in DMEM medium containing 10% heat inactivated serum under a 5% CO_2_ atmosphere. Cells were seeded into 96-well plates at a density of 5 x 10^3^/well with *T*. *cruzi in vitro* derived metacyclic forms (100:1 parasite-host cell ratio). After 4 h of interaction, plates were washed twice with PBS, fresh medium was added, and plates were incubated for 72 h at 37°C (5% CO_2_). After incubation, plates were washed in PBS and cells were fixed for 5 min with cold methanol. Then, plates were washed 3 times with PBS, incubated for at least 30 min in blocking buffer (1% saponin, 2% BSA in PBS), and incubated overnight at 4°C with the anti-TEMA rabbit hyperimmune antiserum (1:2,000 in blocking buffer) (kindly provided by Dr. Victor Tulio Contreras, University of Carabobo, Venezuela), which recognizes the cell body of all major stages of *T*. *cruzi*. Plates were then washed twice with blocking buffer, three times with PBS, and incubated for 60 min at 37°C with a secondary antibody (goat anti-rabbit IgG, Alexa Fluor 488) diluted 1:1,000 in blocking buffer. Finally, the plates were washed five times with PBS, incubated with 100 μl of DAPI (1μg ml^-1^) in PBS, and analyzed in an Operetta High-Content Imaging System (Perkin Elmer, USA) using a 40X high WD objective. Fifty fields per well were imaged using five distinct Z focal planes that were spaced by 1 μm. Maximum projection images (of combined z focal planes) were automatically analyzed using the Harmony High-Content Imaging and Analysis Software (Perkin Elmer, USA). The user-optimized algorithm worked in the following steps: (i) identification of host cell nucleus by DAPI staining, (ii) host cell cytoplasm identification in the digital phase contrast (DPC) channel, (iii) identification of potential intracellular parasites as small spots of DAPI staining in the cytoplasm, and (iv) confirmation of parasite identification by anti-TEMA (Alexa-488) co-staining on small DAPI spots. For each well, the algorithm identified infected and non-infected cells, counted the number of intracellular amastigotes, and calculated the mean number of intracellular parasites per cell (a) and the % of infected cells (b). These data were used to calculate the intracellular parasite growth factor iGF = a x b. The relative intracellular growth (riGF) was calculated using the formula: riGF = iGF_*mutant*_ / iGF_wt_. Growth factor values for each condition represent data from eight replicate wells from ≥ 2 independent experiments. Statistical analysis was performed by ANOVA, which was followed by Tukey-Kramer multiple comparisons test, using GraphPad Prism version 5 software (GraphPad Software Inc, La Jolla, CA, USA).

## Results

### The *T*. *cruzi* AP-1 gamma adaptin (TcAP1-γ) is expressed in all life cycle forms and co-localizes with Golgi and TGN markers

Gamma adaptin is one of the largest subunits of the AP-1 complex. Bioinformatics analysis using the Pfam database (http://pfam.xfam.org/) showed that the *T*. *cruzi* γ adaptin (TcAp1-γ; TcCLB.508257.260; 800 aa, ~ 90 kDa) has the typical structure of eukaryotic γ-adaptins with an N-terminal ‘trunk’ domain (aa 30 to 577) (pfam 01602) and C-terminal appendage or ‘ear’ domain (aa 691 to 798) (pfam 02883), linked by a flexible hinge region. The hinge region of the large γ and β1 adaptins contain sequences that recognize and bind to clathrin [49–51] and the C-terminal ‘ear’ (appendage) domain recruits accessory proteins that modulate AP function to form clathrin-coated vesicles (CCVs) at the TGN [50,52]. By manual sequence analysis we identified a clathrin box candidate in the hinge region of Tcβ1 adaptin (811LFHLE815) that fits the canonical clathrin box motif LΦXΦ(D/E), where Φ is a bulky hydrophobic amino acid and X is any amino acid [53]. We identified two motifs in the hinge region of TcAP-1γ adaptin (627LFEVN630 and 657LFGLK661) that partially resemble the canonical clathrin box.

To study the function of TcAP1-γ, we produced a monoclonal antibody against the hinge domain of this protein (mAb 211.F7) assuming that this region is more accessible than the N- and C-terminal domains, which are likely to be obscured by a myriad of protein-protein interactions (within the AP-1 complex and with accessory and regulatory proteins) [22].

**Fig 1:**
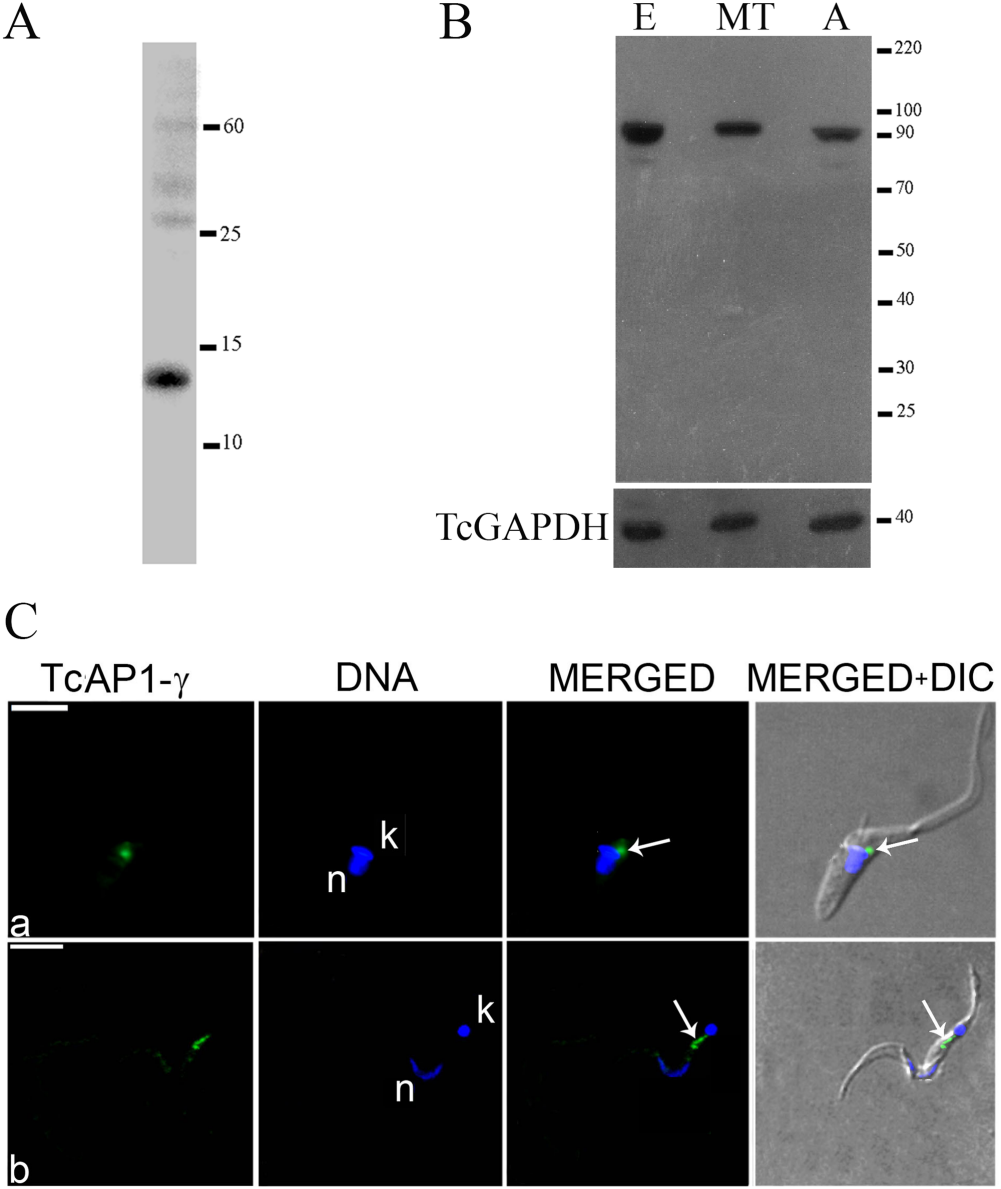
Expression and localization of the *Trypanosoma cruzi* AP-1 complex γ subunit (TcAP1-γ). The purified recombinant protein (histidine-tagged TcAP1-γ hinge domain) (A) and *T*. *cruzi* whole cell extracts (B) were resolved on 10% SDS-PAGE gels, transferred to nitrocellulose membranes and reacted with the anti-TcAP1-γ mAb 211.F7 (produced against the hinge region of TcAP1-γ). E: epimastigotes; MT: metacyclic trypomastigotes; A: amastigotes. An antiserum against the *T*. *cruzi* GAPDH was used as loading control. (C) Parasites were incubated with the 211.F7 mAb (1:80 dilution), followed by incubation with anti-mouse IgG conjugated to Alexa Fluor 488 (1:600 dilution). Nuclear (n) and kinetoplast (k) DNA were stained with Hoechst 33342. (a) Epimastigote and (b) metacyclic trypomastigote. TcAP1-γ labelling is indicated by arrows. DIC, differential interference contrast microscopy. Scale bar = 5μm.

Hybridoma screening after mouse immunization with the histidine-tagged TcAP1-γ hinge domain led to the identification of monoclonal antibody 211.F7 (IgG isotype and kappa positive), which recognizes the recombinant protein (Fig 1A). Analysis of the TcAP1-γ expression pattern during the *T*. *cruzi* life cycle with 211.F7 showed that the TcAP1-γ is expressed in epimastigotes, amastigotes and metacyclic trypomastigotes (Fig 1B).

**Fig 2:**
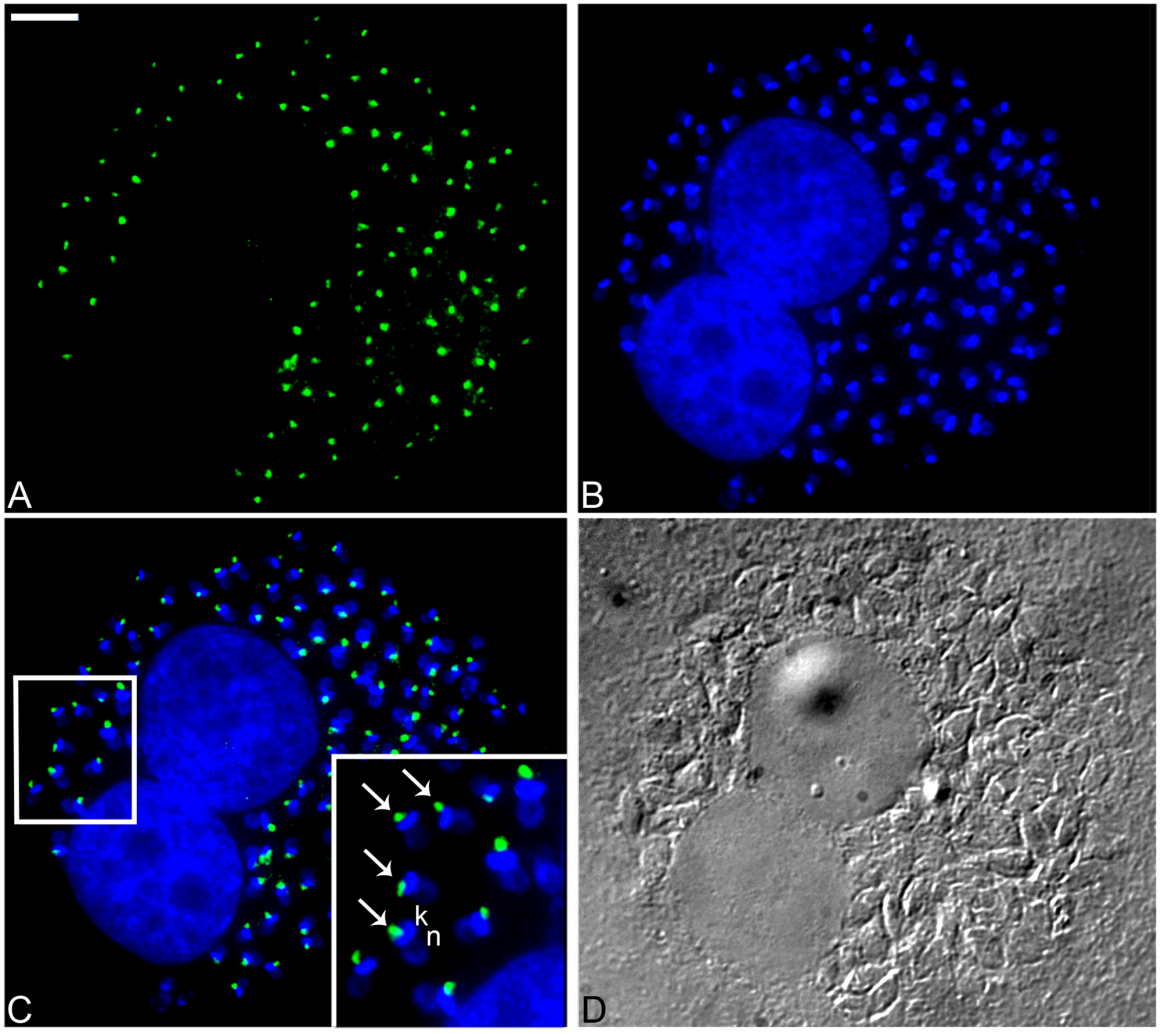
Immunofluorescence localization of the *Trypanosoma cruzi* AP-1 complex γ subunit (TcAP1-γ) in intracellular amastigotes. *T*. *cruzi*-infected VERO cells were labelled with the anti-TcAP1-γ mAb 211.F7, which was detected with anti-mouse IgG conjugated to Alexa Fluor 488. (A) Labeling for TcAP1-γ (Alexa Fluor 488). (B) Host cell and parasite DNA stained with Hoechst 33342, detecting both the nuclear (n) and the kinetoplast (k) DNA of amastigotes. (C) Overlay of A and B, showing the localization of TcAP1-γ (arrow) near the kinetoplast (k). (D) DIC, differential interference contrast microscopy. Scale bar = 10μm.

**Fig 3:**
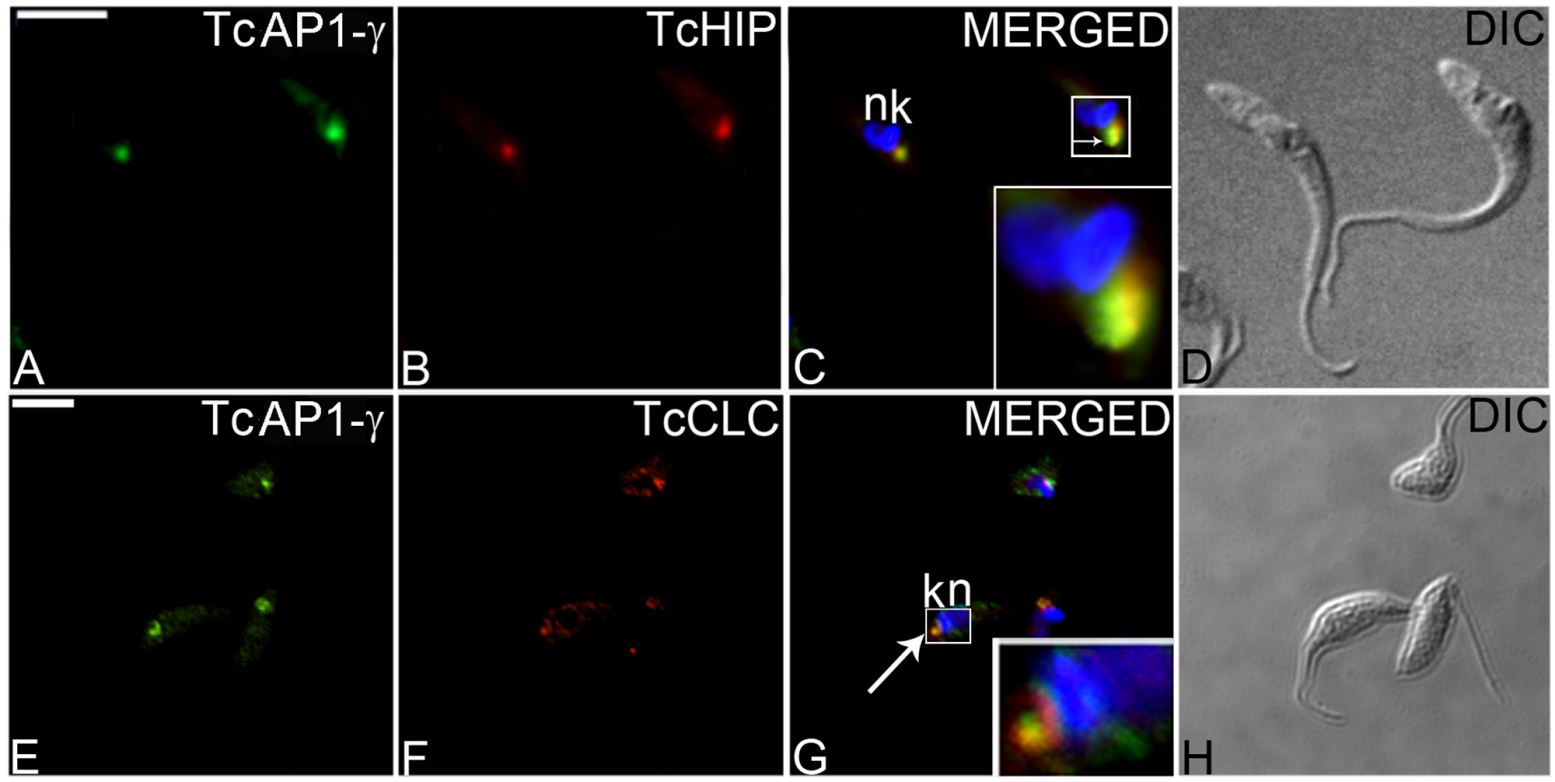
Co-localization of *Trypanosoma cruzi* AP-1 complex γ subunit (TcAP1-γ) with *T*. *cruzi* Golgi markers. Epimastigotes expressing the tagged Golgi marker Huntingtin interacting protein TcHIP-A/C (A-D) or tagged clathrin light chain TcCLC-A/C (E-H) were labelled with an anti-TcAP1-γ mAb 211.F7 that was detected with anti-mouse IgG conjugated to Alexa Fluor 488 followed by incubation with rabbit IgG anti-protein A for TcHIP-A/C and TcCLC-A/C, that were detected with anti-rabbit IgG conjugated to Alexa Fluor 546. Nuclear (n) and kinetoplast (k) DNA were stained with Hoechst 33342. Arrows indicate co-localization. DIC, differential interference contrast microscopy. Scale bar = 5μm.

The AP-1 complex is important for the trafficking of vesicles from the TGN to endosomes [54]. In agreement with this function, immunofluorescence microscopy using the 211.F7 mAb showed that TcAP1-γ was mainly localized in a region adjacent to the kinetoplast in epimastigotes (Fig 1C-a) and intracellular amastigotes (Fig 2) and between the nucleus and the kinetoplast in trypomastigotes (Fig 1C-b), which corresponds to the region where the Golgi complex is located in *T*. *cruzi* [55]. We confirmed that TcAP1-γ was located in the Golgi by co-localization of TcAP1-γ with the Golgi marker Huntingtin interacting protein (TcHIP) [42] (Fig 3A–3C). An interesting result was that TcAp1-γ also co-localized with the clathrin light chain (TcCLC) in the Golgi complex (Fig 3E–3G), suggesting the involvement of both TcAP1-γ and TcCLC in vesicle formation at the TGN.

### *TcAP1-y* gene disruption delays epimastigote growth and decreases metacyclogenesis

**Fig 4:**
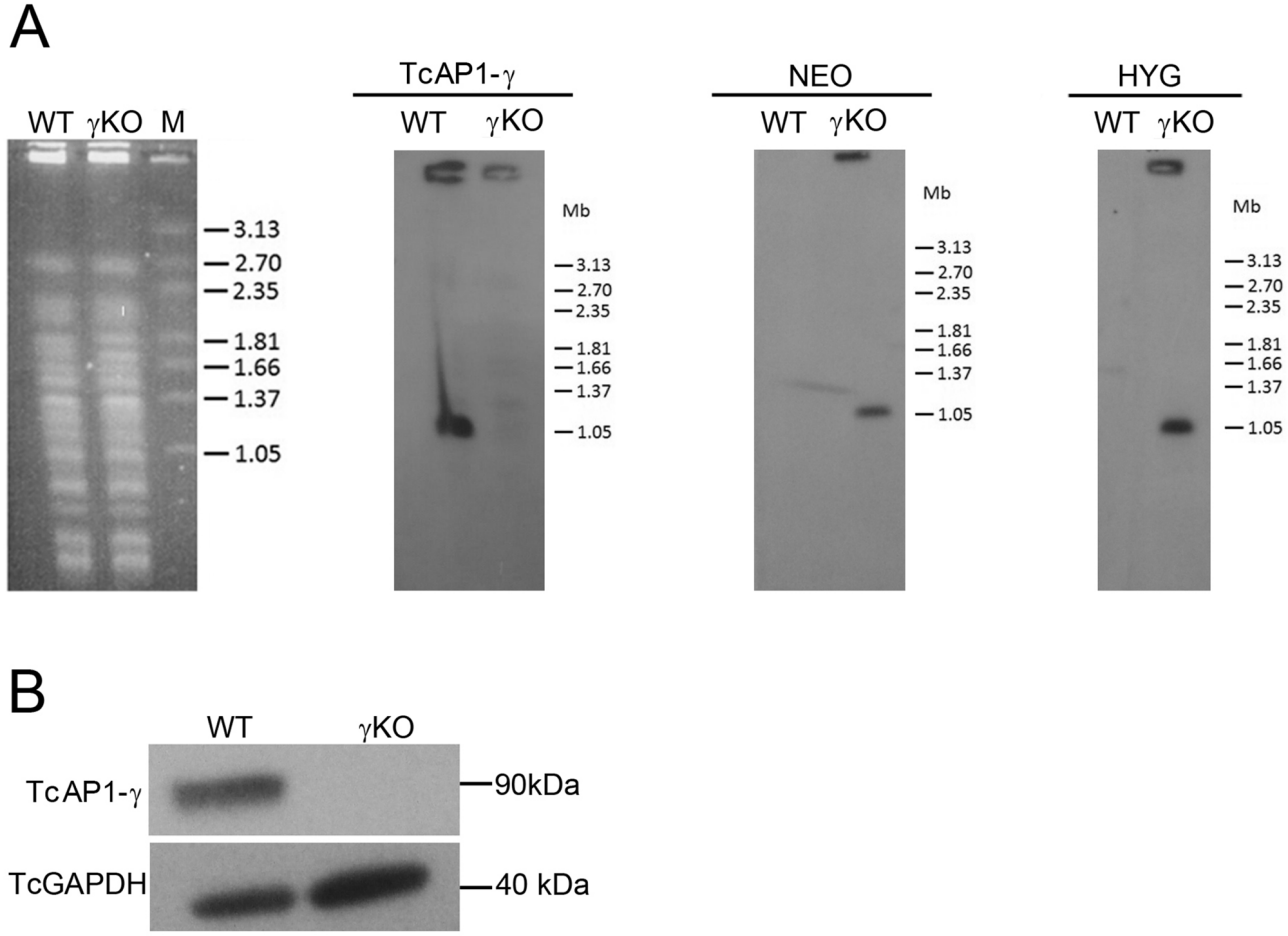
Evaluation of *Trypanosoma cruzi* AP-1 γ subunit (TcAP1-γ) gene knockout. Wild type (WT) and TcAP1-γ knockout (γKO) epimastigotes were analyzed by Southern blot (A) and Western blot (B). (A) High-molecular weight DNA from epimastigotes was separated by PFGE, stained with ethidium bromide (left panel), transferred to nylon membranes and hybridized with [^32^P]-labeled probes corresponding to the *TcAP1-γ*, *NEO* and *HYG* genes. M, molecular mass marker (*Hansenula wingei* chromosomes), in Mb. (B) Epimastigote cell lysates were separated by SDS-PAGE, transferred onto nitrocellulose membranes and incubated with the anti-TcAP1-γ mAb 211.F7 (1:300 dilution). A mouse antiserum anti-TcGAPDH (1:8,000) was used as a loading control. TcAP1-γ protein and gene sequences were detected in WT, but not detected in γKO samples.

To investigate TcAP-1 function in *T*. *cruzi*, we deleted the TcAP1-γ gene in epimastigotes by replacing both alleles with resistance markers *NEO* and *HYG* (S1 Fig). *TcAP1-γ* gene knockout was confirmed by Southern blot from PFGE of chromosomes from TcγKO parasites (Fig 4A). The *TcAP1-γ* radioactive probe recognized a 1.05 Mb chromosomal band only in WT parasites, but not in TcγKO parasites (γKO) (Fig 4A), indicating that the *TcAP1-γ* gene was deleted from the genome in TcγKO parasites. Additionally, *NEO* and *HYG* probes only hybridized with the 1.05Mb chromosomal band in the TcγKO parasites (Fig 4A), indicating that the NEO and HYG cassettes were correctly integrated into the *TcAP-1γ* locus. Western blot analysis with total protein extracts from WT and TcγKO parasites using the 211.F7 mAb showed that TcAP1-γ was not detected in TcγKO parasites, but it was readily detected in extracts of WT epimastigotes (Fig 4B), confirming the ablation of TcAP1-γ expression.

**Fig 5:**
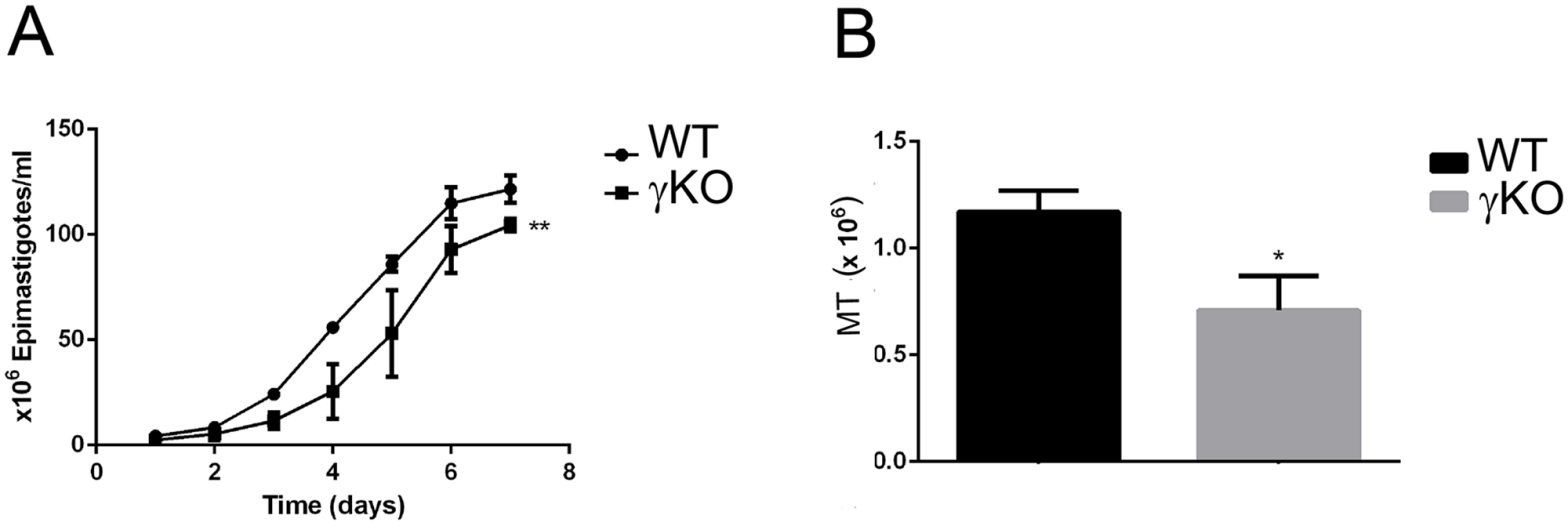
Effect of *Trypanosoma cruzi* AP-1 subunit γ (TcAP1-γ) gene knockout on epimastigote proliferation and differentiation into metacyclic trypomastigotes. (A) Growth curves of wild-type (WT, circles) and TcγKO (γKO, squares) epimastigotes. Data represent mean ± SD of three independent experiments. ** p < 0.05 (test-t). (B) Number of metacyclic trypomastigotes (MT) obtained after 72 h of metacyclogenesis (epimastigote to metacyclic trypomastigote differentiation) *in vitro* for WT (black column) and γKO (gray column) parasite populations. Data represent mean ± SD of three independent experiments. * P < 0.01 (test-t).

TcAP1-γ null mutant parasites were viable; however, growth curves of WT and TcγKO epimastigotes showed that null mutant populations grew significantly slower than wild-type cells (Fig 5A). When TcγKO epimastigotes were incubated in TAU3AAG medium (to induce metacyclogenesis *in vitro*), the ability of KO parasites to undergo differentiation into metacyclic trypomastigotes was approximately 40% lower than that of WT epimastigotes (Fig 5B). These results show that lack of TcAP1-γ significantly decreases parasite proliferation and differentiation into infective forms *in vitro*.

### TcAP1-γ knockout disturbs the maturation and targeting of cruzipain

**Fig 6:**
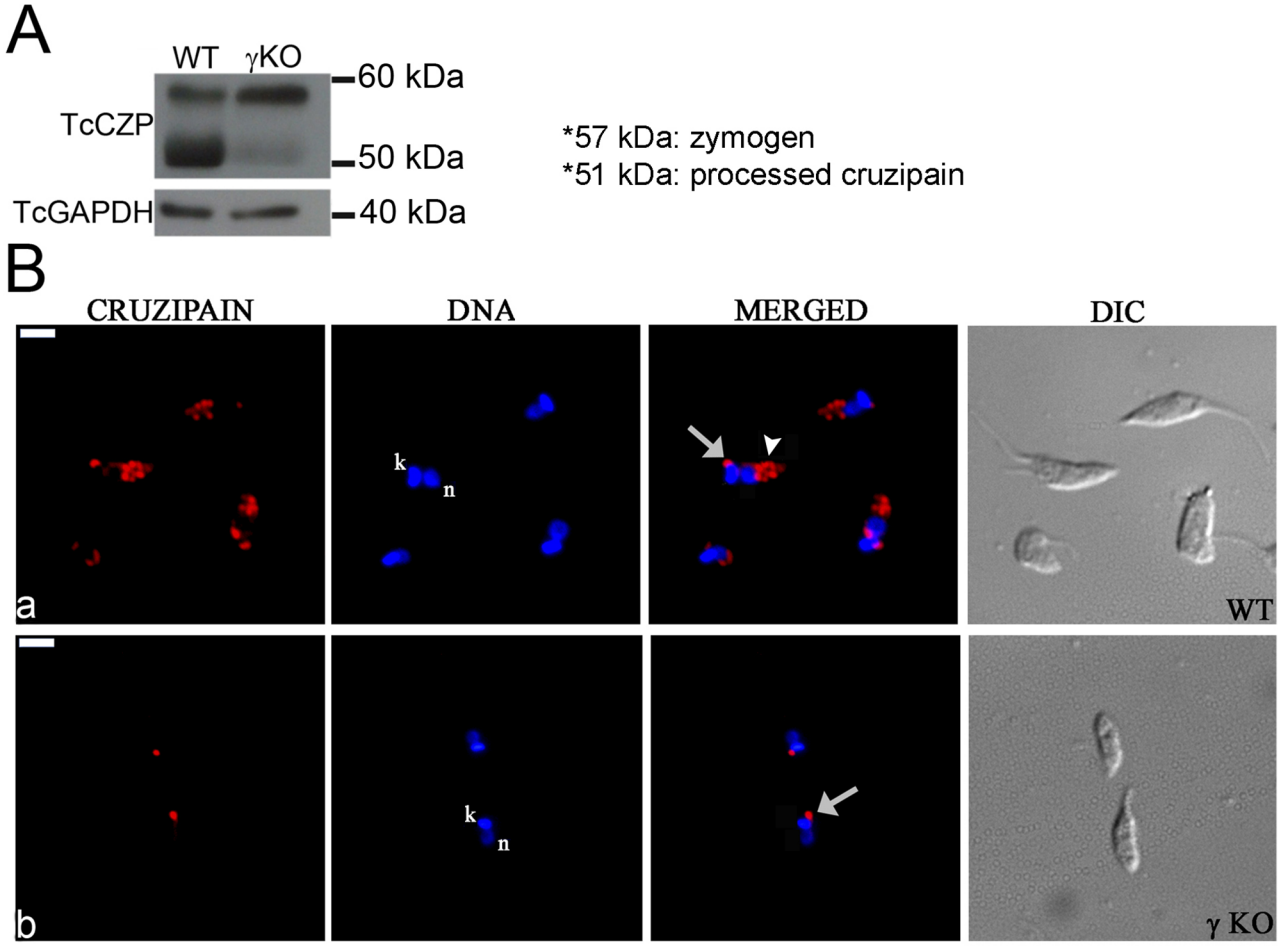
Effect of *Trypanosoma cruzi* AP-1 γ subunit (TcAP1-γ) gene knockout on cruzipain expression and localization. (A) Whole cell lysates from wild-type (WT) and TcγKO (γ KO) epimastigotes were separated by SDS-PAGE, transferred to nitrocellulose membranes and labeled with an anti-cruzipain (TcCZP) antiserum. In γKO epimastigotes, the levels of the zymogen form (57 kDa) of TcCZP were increased, while the levels of the mature enzyme (51 kDa) were decreased (to a faint band) compared with the WT. Labeling for the *T*. *cruzi* GAPDH was used as a loading control. (B) Wild-type (WT) and AP-1 γ null mutant (γ KO) epimastigotes were labeled with an anti-cruzipain antiserum and detected with anti-mouse IgG conjugated to Alexa Fluor 594. Cruzipain localized in the region of the Golgi complex (arrows) and reservosomes (arrowheads) in WT (**a)**, but it appeared absent from reservosomes in the γ null mutant (b). Nuclear (n) and kinetoplast (k) DNA were stained with Hoechst 33342. DIC, differential interference contrast microscopy. Scale bar = 5μm.

Cruzipain is the major cysteine proteinase in *T*. *cruzi* and a key enzyme for nutrition, differentiation and for the establishment of host cell infection [13,56]. This hydrolase is synthesized as a zymogen of 57 kDa, from which the N-terminal pro-domain is removed autocatalytically, to generate the active enzyme (51 kDa) [57]. Once cruzipain is processed in the endoplasmic reticulum/Golgi complex, it is delivered to reservosomes, which only contain mature cruzipain [40]. Given the potential importance of TcAP1-γ in vesicular traffic from the TGN—including reservosome-bound traffic in *T*. *cruzi*–we investigated whether the transport of cruzipain to reservosomes was affected in TcγKO parasites. First we analyzed the expression of cruzipain in TcγKO cells using an anti-cruzipain antiserum that recognizes both the unprocessed and mature form of this enzyme [40]. Western blot analysis showed that the cruzipain zymogen form (57 kDa) was recognized by the anti-cruzipain antiserum in both WT and in TcγKO epimastigotes, although the latter had increased zymogen levels (Fig 6A). In contrast, the levels of the mature form of cruzipain (51 kDa) were clearly reduced in TcγKO cells where only a faint band corresponding to mature cruzipain was detected (Fig 6A). These data suggest that the processing of the cruzipain zymogen into mature cruzipain decreased substantially in the TcγKO mutants.

Since TcγKO parasites exhibited decreased processing of cruzipain into its mature form, we examined whether TcAP1-γ deletion affected the transport of cruzipain to reservosomes, which contain only mature cruzipain in WT epimastigotes. Indeed, immunofluorescence analysis showed that cruzipain was not detected in the reservosomes of TcγKO epimastigotes (Fig 6B). To confirm that the absence of cruzipain in the reservosomes of TcγKO parasites was due the lack of TcAP1-γ expression, we transfected TcγKO parasites with an episomal construct constitutively expressing TcAP1-γ. Western blot analysis confirmed that TcAP1-γ was highly expressed in the complemented TcγKO(pTc-AP1γ) parasites and immunofluorescence analysis showed that the TcAP1-γ expressed in complemented parasites was correctly addressed to the Golgi region (close to kinetoplast) (Figs A and B in S3 Fig). The complemented parasite growth was lower than the WT parasites, however the ability to differentiate to metacyclic trypomastigote forms was higher than the TcγKO cells and comparable to the WT strain (Figs C and D in S3 Fig). Complementation of TcAP1-γ expression in γKO mutants restored maturation and cruzipain localization to the reservosomes (S4 Fig), implicating the AP-1 complex in the formation of reservosome-bound vesicles containing cruzipain, at the TGN.

We further investigated whether cruzipain was targeted to the cell surface in γKO amastigotes because intense cruzipain labeling was observed in the surface of cell-derived amastigotes [58]. Immunofluorescence assays showed that the intensity of cruzipain labeling was highly reduced in the surface of γKO amastigotes compared to WT amastigotes (S5 Fig).

### TcγKO trypomastigotes have decreased infectivity towards mammalian host cells

Cruzipain is secreted to the environment by trypomastigotes, it is also present in the amastigote surface, which is associated with increased parasite potential to infect and develop within the host cells [59,60]. Given that *T*. *cruzi* γKO parasites had a clear defect in cruzipain processing, we hypothesized that this mutant may have reduced ability to infect host cells. To test this hypothesis, we quantified the infectivity of WT, TcγKO and TcγKO(pTc-AP1γ) parasites towards HeLa and VERO cells (as representatives of epithelial and fibroblast cell lineages, respectively) using a high-content imaging platform with parameters optimized for the high-throughput analysis of amastigote infection in many cells (>5,000) per well.

**Fig 7:**
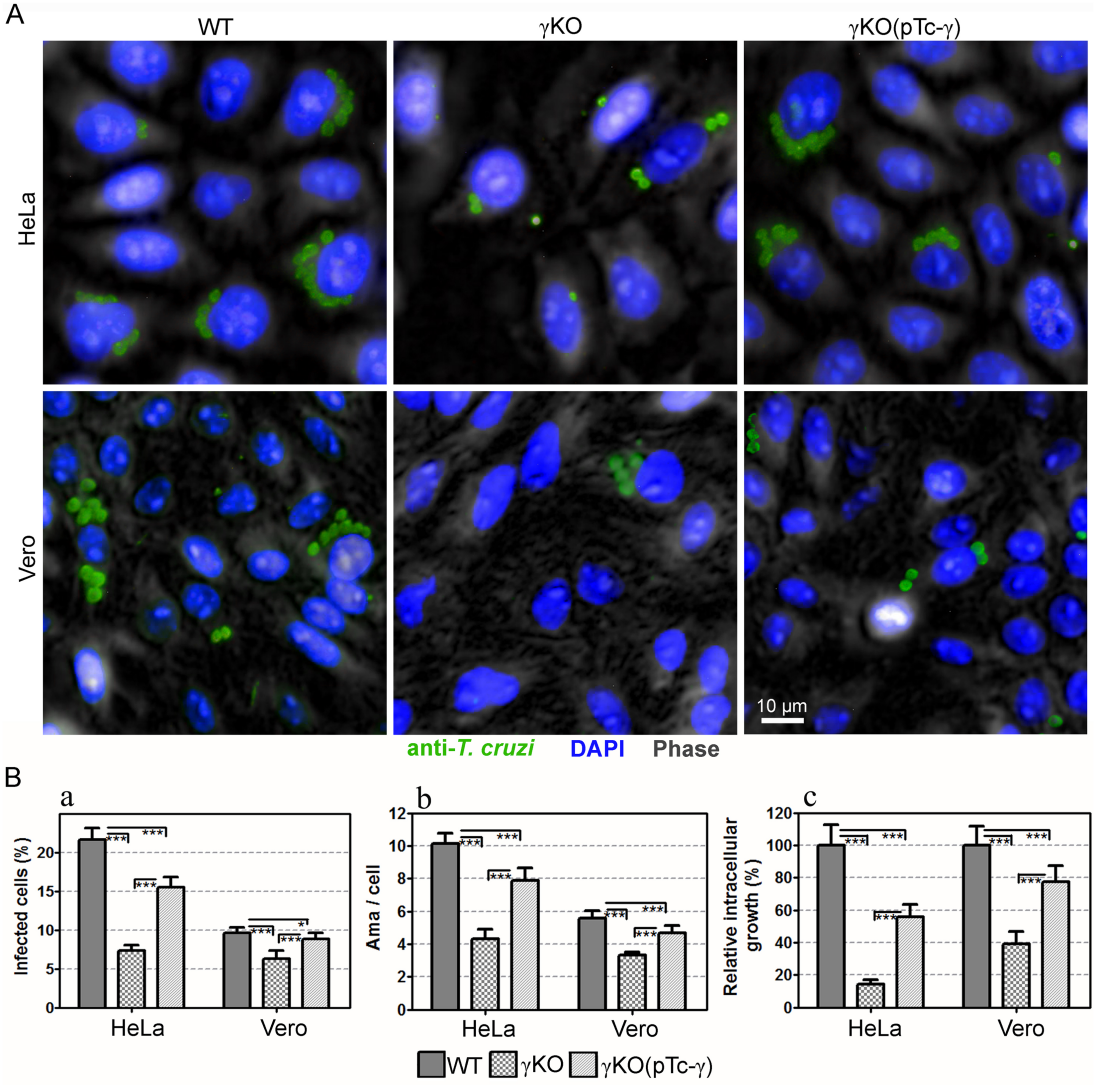
Effect of *Trypanosoma cruzi* AP-1 γ subunit (TcAP1-γ) gene knockout on *T*. *cruzi* γKO infection in mammalian cells. Vero and HeLa cells were infected with WT, TcγKO (γKO) and TcγKO(pTc-γ) (complemented TcγKO) metacyclic trypomastigotes for 72 h. Then, they were labeled with the anti-*T*. *cruzi* serum (anti-TEMA) (detected using anti-rabbit IgG conjugated to Alexa Fluor 488) and DAPI (for DNA detection). Cells were imaged using the Operetta Imaging System (PerkinElmer) and analyzed using the Harmony High Content Imaging and Analysis Software (Perkin Elmer). (A) Immunofluorescence images of host cells infected with intracellular amastigotes. (B) Infection quantification was performed using the Harmony High-Content Imaging and Analysis Software, which calculated the percentage of infect cells (a, left graph), mean number of amastigotes/cell (b, middle graph), and relative intracellular growth (riGF = iGF_*mutant*_ / iGF_*WT*_, where iGF = a x b) for ≥5000 cells/well (8 wells/experimental condition). Data represent the mean and SD of one representative experiment. ***p < 0.001, **p < 0.01 and *p < 0.05.

Our data showed that knockout of AP-1γ led to a statistically significant decrease in *T*. *cruzi* infection in both VERO and in HeLa cells (Fig 7A and 7B). The percentage of infected cells decreased from 21.3% to 7.4% (p<0.001) and 9.6% to 6.3% (p<0.001) in HeLa and VERO cells, respectively (Fig 7B, left panel). The mean number of intracellular amastigotes per infected cell also significantly decreased, from 10.1 to 4.3 (p<0.001) and 5.6 to 3.3 (p<0.001), in HeLa and VERO cells, respectively (Fig 7B, middle panel).

Complementation of TcγKO parasites with an episomal construct expressing TcAP1-γ was sufficient to significantly restore the infection ability of TcγKO towards both mammalian cell lines; however, complementation did not fully restore parasite infectivity compared with WT parasites (Fig 7B, right panel). Trypomastigote release (by host cell bursting) was also strongly decreased (and delayed) in cultures infected with TcγKO (< 10% of the trypomastigote production of cells infected with WT parasites).

### The structure of the Golgi complex and reservosomes is preserved in *T*. *cruzi* γKO epimastigotes

Since the TcAP1-γ knockout impairs the transport of cruzipain from TGN, we investigated whether the accumulation of the unprocessed form of this protease could cause morphological alterations in the Golgi complex of TcγKO epimastigotes. Transmission electron microscopy analysis indicated that the Golgi complex of TcγKO epimastigotes had no noteworthy cisternae alterations (Figures A and B in S6 Fig), although the cisternae were more contrasted and easily detected. Once Golgi-derived vesicles are involved in the reservosome formation, we also examined whether reservosomes were present in TcγKO cells. Indeed, ultrastructural analysis of TcγKO epimastigotes showed the presence of reservosomes at the posterior region of these parasites (Figures C and D in S6 Fig).

## Discussion

The AP-1 complex is responsible for vesicle trafficking from the TGN to endosomes in eukaryotes. Within the Trypanosomatidae, the AP-1 complex is essential in *T*. *brucei* and required for infectivity in *Leishmania* [30,31]. Nevertheless, the function of the AP-1 complex in the Chagas disease agent *T*. *cruzi* has not been examined to date. In this work we show that AP1-γ adaptin is expressed in different *T*. *cruzi* life stages, including parasite forms found in the invertebrate vector (epimastigotes), metacyclic trypomastigotes (responsible for initial host infection), and the replicating intracellular stage (amastigotes).

Production of the monoclonal antibody 211.F7, which targets the hinge region of TcAP1-γ, allowed us to determine that this protein localizes to the Golgi complex, as confirmed by co-localization with the Golgi marker TcHIP, a palmitoyl acyl transferase [42]. TcAP1-γ also partially co-localized with the clathrin light chain (TcCLC), which is found (albeit not exclusively) in the TGN [43]. Recently, we showed that clathrin interacts with both AP-1 and AP-4 complexes in a clathrin interactome study [61]. These data agree with the results of the present study and suggest that an AP-1/clathrin machinery operates at the TGN, in *T*. *cruzi*.

Since AP-1 is responsible for addressing proteases to lysosomes [19,27], we investigated whether AP-1 was involved in the transport of the major protease cruzipain to the reservosomes, the lysosome-related organelle of *T*. *cruzi* epimastigotes. Cruzipain is a differentially glycoprotein expressed during the *T*. *cruzi* life cycle, it has higher activity in epimastigotes, where it is found in the Golgi as a pre-proprotein [16,62]. Cruzipain is then targeted to reservosomes independent from the addition of mannose-6-phosphate (M6P) residues [3,10], which target lysosomal hydrolases to endosomes in other eukaryotes [53]. The M6P biosynthetic pathway is absent in trypanosomes [28,29], which must, therefore, rely on alternative mechanisms of lysosomal hydrolase targeting. Our data strongly suggest a direct role for the AP-1 in cruzipain trafficking because ablation of TcAP1-γ leads to retention of cruzipain in the Golgi complex. Cruzipain was mostly found in its unprocessed form (57 kDa) in TcγKO epimastigotes, indicating that maturation of cruzipain occurs during its transport to the reservosomes, although it could also occur (to some degree) in the Golgi complex, as revealed by the small fraction of the mature form of this enzyme (51 kDa) detected in TcγKO parasites. As expected, complementation of TcγKO with a plasmid expressing TcAP1-γ restored the transport of cruzipain to the reservosomes. Interestingly, the AP-1/clathrin-machinery is necessary for the transporting the cysteine protease trypanopain to the lysosome in *T*. *brucei* procyclic forms, but it is dispensable in bloodstream forms [31].

Ablation of TcAP1-γ was not lethal, unlike that observed in *T*. *brucei* where TbAP1-γ is essential [31]. However, proliferation of TcγKO epimastigotes was reduced, as well as the efficiency of metacyclogenesis. It is possible that *T*. *cruzi* AP-1 plays a more defined role by mediating the transport of a reduced number of cargos, including cruzipain, to reservosomes. Therefore, disruption of this complex would not impair the overall metabolism of the parasite and biogenesis and full activity of reservosomes. In the absence of the specific cargos that depend on AP-1 for targeting to reservosomes, the endocytosed material in this organelle would not be fully degraded for use as an energy source by epimastigotes, reducing cell proliferation and the ability of epimastigotes to differentiate into trypomastigotes.

Reservosomes are formed by the fusion of endocytic vesicles from the flagellar pocket and cytostome with Golgi-derived vesicles. Electron microscopy analysis suggested that TcγKO parasites contain reservosomes, indicating that the biogenesis of these organelles was not fully impaired by TcAP1-γ ablation. Our results agree with a previous study showing that cruzipain inhibitors reduce *T*. *cruzi* growth and differentiation [56], implicating the lack of active cruzipain as a relevant consequence of TcAP1-γ knockout. However, our findings differ from those reported by Engel and co-workers [17], who showed that inhibition of cruzipain activity in epimastigotes led to cell death due to the accumulation of cruzipain in the Golgi, which was followed by large alterations in Golgi membranes. TcγKO epimastigotes in our study were viable and cisternae abnormalities in the Golgi complex were not observed, but the Golgi complex cisternae of TcγKO parasites were more contrasted and easily detected by transmission electron microscopy.

Although TcAP1-γ ablation only marginally affected the growth rate of epimastigote forms, it strongly impaired the ability of *T*. *cruzi* to infect host cells from two distinct cell lineages. The reduced number of infected host cells and the small number of intracellular amastigotes observed after 72 h of infection suggest that invasion and/or amastigote proliferation were affected in TcγKO parasites. Furthermore, the number of trypomastigotes released from infected cells was considerably reduced compared with that observed in cultures infected with WT parasites. We hypothesize that the major cause of these defects was the retention of cruzipain in the Golgi complex. Although this protease is expressed at lower levels in trypomastigotes and amastigotes, compared with epimastigotes [63], cruzipain is critical for trypomastigote infectivity and for intracellular amastigote development [13,56]. Some isoforms of cruzipain are secreted by trypomastigotes [59,64] and they play an important role as virulence factors in Chagas disease [59,62]. Additionally, a striking observation is the presence of cruzipain on the surface of amastigotes [58,65], which may play an important role in parasite survival within host cells. Interestingly, in the γKO amastigotes the cruzipain expression at the cell surface was reduced.

We observed that the ability of TcγKO parasites to infect host cells was only partially restored by complementation with the episomal expression of TcAP1-γ. This result could be explained by non-uniform episomal TcAP1-γ expression within the complemented parasite population, where the maturation and transport of cruzipain in the cells expressing lower levels of TcAP1-γ levels may not have been fully restored.

In addition, we observed that whereas the metacyclogenesis rate was fully restored in the complementing strain, the growth rate of the TcγKO epimastigotes was apparently not affected by the episomal expression of TcAP1-γ. One possible reason is that the complemented TcγKO epimastigotes were grown in LIT medium supplemented with the antibiotic blasticidin to avoid the loss of the plasmid pTcGW-AP-1γ-bsd during cell proliferation.

Taken together, our results show that AP-1 machinery mediates post-Golgi sorting of cruzipain to reservosomes in *T*. *cruzi* and that AP-1 function is important for parasite infectivity towards host cells. Further studies are necessary to determine the other cargos that depend on AP-1 for transport within the *T*. *cruzi* endosomal system.

## Supporting information

S1 Fig Strategy of *TcAP1-γ* gene knockout. A) Schemes of the plasmids pTc2KO-AP-1γ-neo and pTc2KO-AP-1γ-hyg, designed to replace the two alleles of the TcAP1-γ gene, by homologous recombination for replacement with the resistance markers NEO and HYG, as depicted in B.
(TIF)

S2 Fig Diagram of the plasmids used for complementation of TcγKO parasites. The basic structure of pTcGW 1.1 Gateway expression vectors (Kugeratski et al., 2015) were used as a backbone to construct the plasmid pTcGW-bsd (A) for Tcγ cloning. pTcGW-bsd contains the *T*. *cruzi* Dm28c ribosomal promoter and three distinct *T*. *cruzi* intergenic regions (IRs) that contain the following sequence elements for the correct processing of the transcripts of the cloned genes into the plasmid: IR1 (IR from *T*. *cruzi* ubiquitin locus, 278 bp), IR2 (IR between the *T*. *cruzi* genes TcCLB.504069.70 and TcCLB. 504069.80, 421 bp) and IR3 (IR between the *T*. *cruzi* genes TcCLB.506295.100 and TcCLB.506295.110, 482 bp). This plasmid also contains a selectable marker (Blasticidin resistance gene, BSD), and two recombination sites (*att*R1 and attR2) flanking the *ccdB* gene, for negative selection in *E*. *coli*. The Tcγ gene was amplified by PCR using primers containing the *att*B recombination sites (Table 1) and subcloned (by recombination) into *att*R1 and *att*R2 of pTcGW-bsd, resulting in the pTcGW-AP1γ-bsd plasmid (B).
(TIF)

S3 Fig Ectopic expression of TcAP1-γ in TcγKO epimastigotes. Wild-type (WT), TcAP1-γ null mutant (γKO) and TcAP1-γ-complemented TcγKO [γKO(pTc-γ)] epimastigotes were analyzed for Western blot expression (A) and, localization of TcAP1-γ by immunofluorescence microscopy (B), using the anti-TcAP1-γ mAb 211.F7 as well as the abilities for proliferation (C) and differentiation (D). **A)** Western blot analysis using whole cell lysates. TcAP1-γ was not detected in γKO parasites, but was highly expressed in the complemented γKO(pTc-γ) cells, compared with WT parasites. An antiserum against GAPDH of *T*. *cruzi* was used as a loading control. **B)** Immunolocalization of TcAP1-γ. The anti-TcAP1-γ mAb 211.F7 (1:80 dilution) was detected with an anti-mouse IgG conjugated to Alexa Fluor 594 (1:600 dilution). In the complemented γKO(pTcγ) parasite, TcAP1-γ is localized near the kinetoplast as in control WT cells (arrows), indicating that the overexpressed TcAP1-γ is correctly addressed to the Golgi. Nuclear (n) and kinetoplast (k) DNA were stained with Hoechst 33342. DIC, differential interference contrast microscopy. Scale bars = 5μm. **C)** Growth curves of wild-type (WT, circles) and complemented γKO(pTcγ) (squares) epimastigotes. Data represent the mean ± SD of three independent experiments. ** p < 0.05 (test-t). **D)** Number of metacyclic trypomastigotes (MT) obtained after 72 h of metacyclogenesis (epimastigote to metacyclic trypomastigote differentiation) *in vitro*, for the WT (black column) and the complemented γKO(pTcγ) (gray column) parasite populations. Data represent mean ± SD of three independent experiments. * P < 0.01 (test-t).
(TIF)

S4 Fig The cruzipain maturation and reservosome targeting are restored in the complemented γKO(pTcγ) strain. **A)** Whole cell lysates from wild-type (WT) and the complemented γKO(pTcγ) epimastigotes were separated by SDS-PAGE, transferred to nitrocellulose membranes and labeled with an anti-cruzipain (TcCZP) antiserum. In γKO(pTcγ) epimastigotes, the processing of cruzipain was restored as observed by the presence of a band of 51 kDa corresponding to the mature form of the enzyme. Labeling for the *T*. *cruzi* GAPDH was used as a loading control. **B)** Wild-type (WT) and γKO(pTcγ) epimastigotes were labeled with an anti-cruzipain antiserum, detected with anti-mouse IgG conjugated to Alexa Fluor 594. Cruzipain localized in the region of the Golgi complex (arrows) and reservosomes (arrowheads) in WT and the complemented strain. Nuclear (n) and kinetoplast (k) DNA were stained with Hoechst 33342. DIC, differential interference contrast microscopy. Scale bar = 5μm
(TIF)

S5 Fig Effect of *Trypanosoma cruzi* AP-1gamma subunit (TcAP1-γ) gene knockout on cruzipain localization in amastigote surface. Wild-type (WT) and AP-1 γ null mutant (γKO) amastigote (not permeabilized) were labelled with anti-cruzipain antiserum and, detected with anti-mouse IgG conjugated to Alexa Fluor 594. A strong labelling was localized in the WT surface (A), whereas the γKO amastigote surface was faintly labelled (B). Nuclear (n) and kinetoplast (k) DNA were stained with Hoechst 33342. DIC, differential interference contrast microscopy. Scale bar = 5μm
(TIF)

S6 Fig Ultrastructural analysis of *T*. *cruzi* epimastigote forms from wild-type (WT) and TcAP1-γ knockout (TcγKO) parasites. The Golgi complex (arrow) is observed at the anterior region of both WT (**a**) and TcγKO (**b**) parasites. No noticeable morphological alteration is observed, except that the Golgi cisternae are more contrasted and easily detected in TcγKO parasites. Reservosomes (*) with electron-dense matrix are found at the cell posterior region in both WT (**c**) and TcγKO (**d**) parasites, with no remarkable difference in size, shape and density. N = nucleus, K = kinetoplast.
(TIF)
