# Supplementary figures and images for: Knockout of the gamma subunit of the AP-1 adaptor complex in the human parasite Trypanosoma cruzi impairs infectivity and differentiation and prevents the maturation and targeting of the major protease cruzipain
Source: PLoS One. 2017 Jul 31;12(7):e0179615. doi: 10.1371/journal.pone.0179615 (PMC5536268; doi:10.1371/journal.pone.0179615)

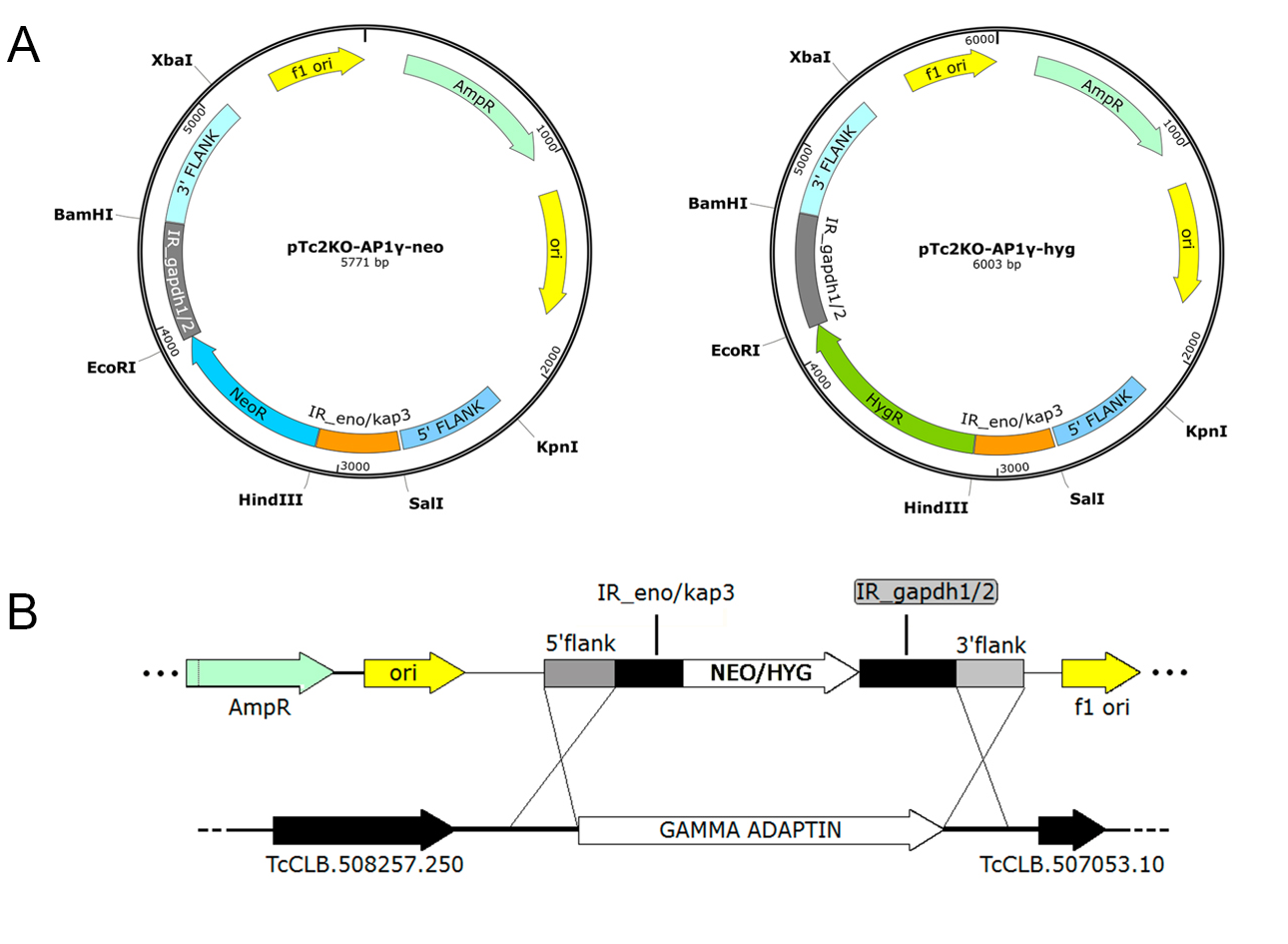

Supplement: S1 Fig — A) Schemes of the plasmids pTc2KO-AP-1γ-neo and pTc2KO-AP-1γ-hyg, designed to replace the two alleles of the TcAP1-γ gene, by homologous recombination for replacement with the resistance markers NEO and HYG, as depicted in B. (TIF) [file pone.0179615.s001.tif]

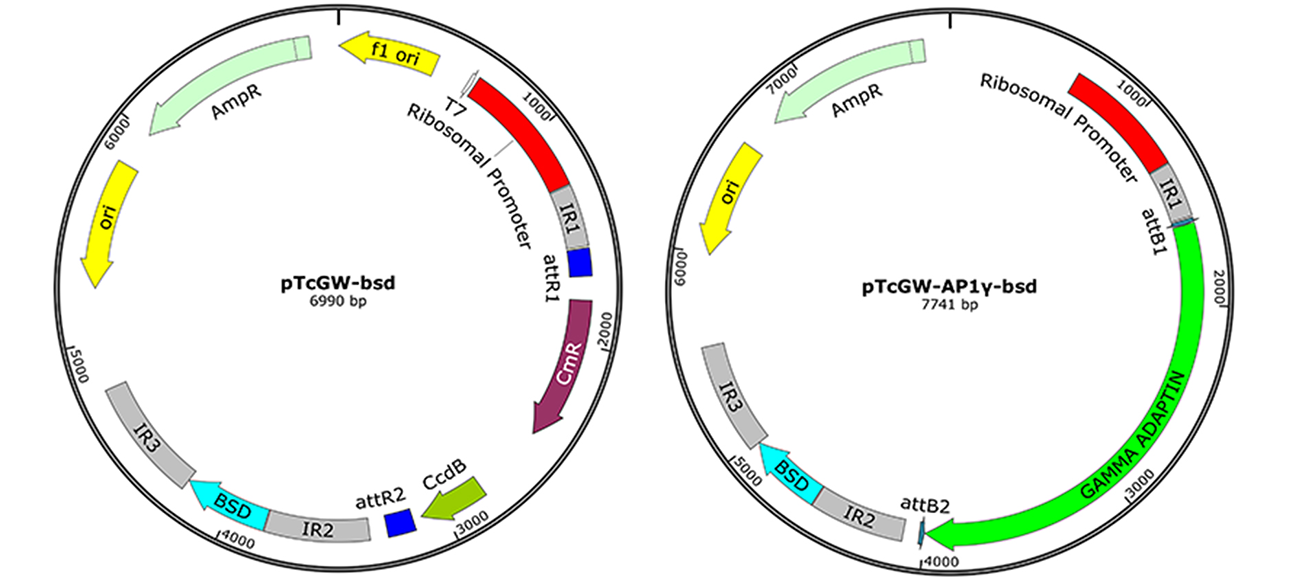

Supplement: S2 Fig — The basic structure of pTcGW 1.1 Gateway expression vectors (Kugeratski et al., 2015) were used as a backbone to construct the plasmid pTcGW-bsd (A) for Tcγ cloning. pTcGW-bsd contains the T. cruzi Dm28c ribosomal promoter and three distinct T. cruzi intergenic regions (IRs) that contain the following sequence elements for the correct processing of the transcripts of the cloned genes into the plasmid: IR1 (IR from T. cruzi ubiquitin locus, 278 bp), IR2 (IR between the T. cruzi genes TcCLB.504069.70 and TcCLB. 504069.80, 421 bp) and IR3 (IR between the T. cruzi genes TcCLB.506295.100 and TcCLB.506295.110, 482 bp). This plasmid also contains a selectable marker (Blasticidin resistance gene, BSD), and two recombination sites (attR1 and attR2) flanking the ccdB gene, for negative selection in E. coli. The Tcγ gene was amplified by PCR using primers containing the attB recombination sites (Table 1) and subcloned (by recombination) into attR1 and attR2 of pTcGW-bsd, resulting in the pTcGW-AP1γ-bsd plasmid (B). (TIF) [file pone.0179615.s002.tif]

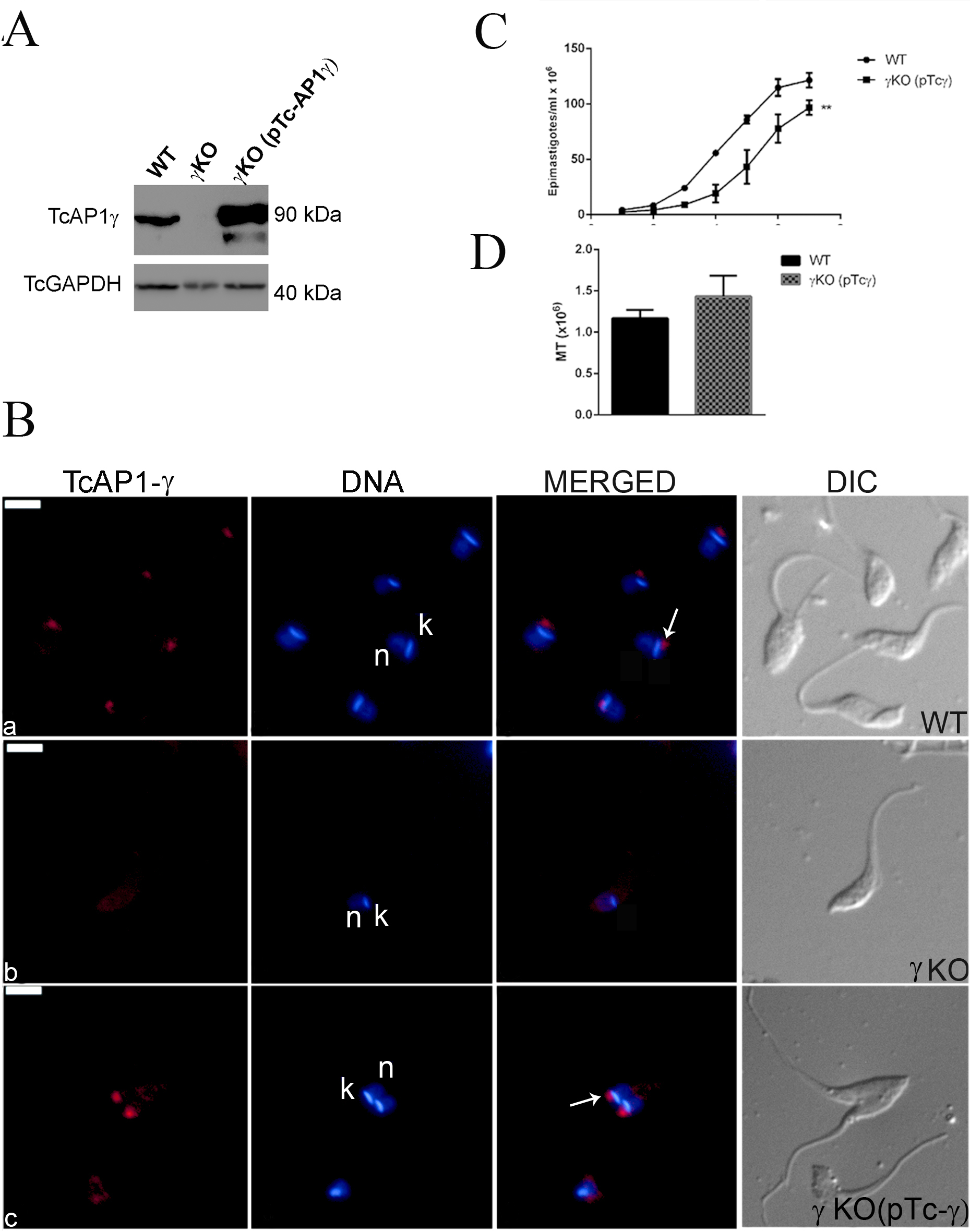

Supplement: S3 Fig — Wild-type (WT), TcAP1-γ null mutant (γKO) and TcAP1-γ-complemented TcγKO [γKO(pTc-γ)] epimastigotes were analyzed for Western blot expression (A) and, localization of TcAP1-γ by immunofluorescence microscopy (B), using the anti-TcAP1-γ mAb 211.F7 as well as the abilities for proliferation (C) and differentiation (D). A) Western blot analysis using whole cell lysates. TcAP1-γ was not detected in γKO parasites, but was highly expressed in the complemented γKO(pTc-γ) cells, compared with WT parasites. An antiserum against GAPDH of T. cruzi was used as a loading control. B) Immunolocalization of TcAP1-γ. The anti-TcAP1-γ mAb 211.F7 (1:80 dilution) was detected with an anti-mouse IgG conjugated to Alexa Fluor 594 (1:600 dilution). In the complemented γKO(pTcγ) parasite, TcAP1-γ is localized near the kinetoplast as in control WT cells (arrows), indicating that the overexpressed TcAP1-γ is correctly addressed to the Golgi. Nuclear (n) and kinetoplast (k) DNA were stained with Hoechst 33342. DIC, differential interference contrast microscopy. Scale bars = 5μm. C) Growth curves of wild-type (WT, circles) and complemented γKO(pTcγ) (squares) epimastigotes. Data represent the mean ± SD of three independent experiments. ** p < 0.05 (test-t). D) Number of metacyclic trypomastigotes (MT) obtained after 72 h of metacyclogenesis (epimastigote to metacyclic trypomastigote differentiation) in vitro, for the WT (black column) and the complemented γKO(pTcγ) (gray column) parasite populations. Data represent mean ± SD of three independent experiments. * P < 0.01 (test-t). (TIF) [file pone.0179615.s003.tif]

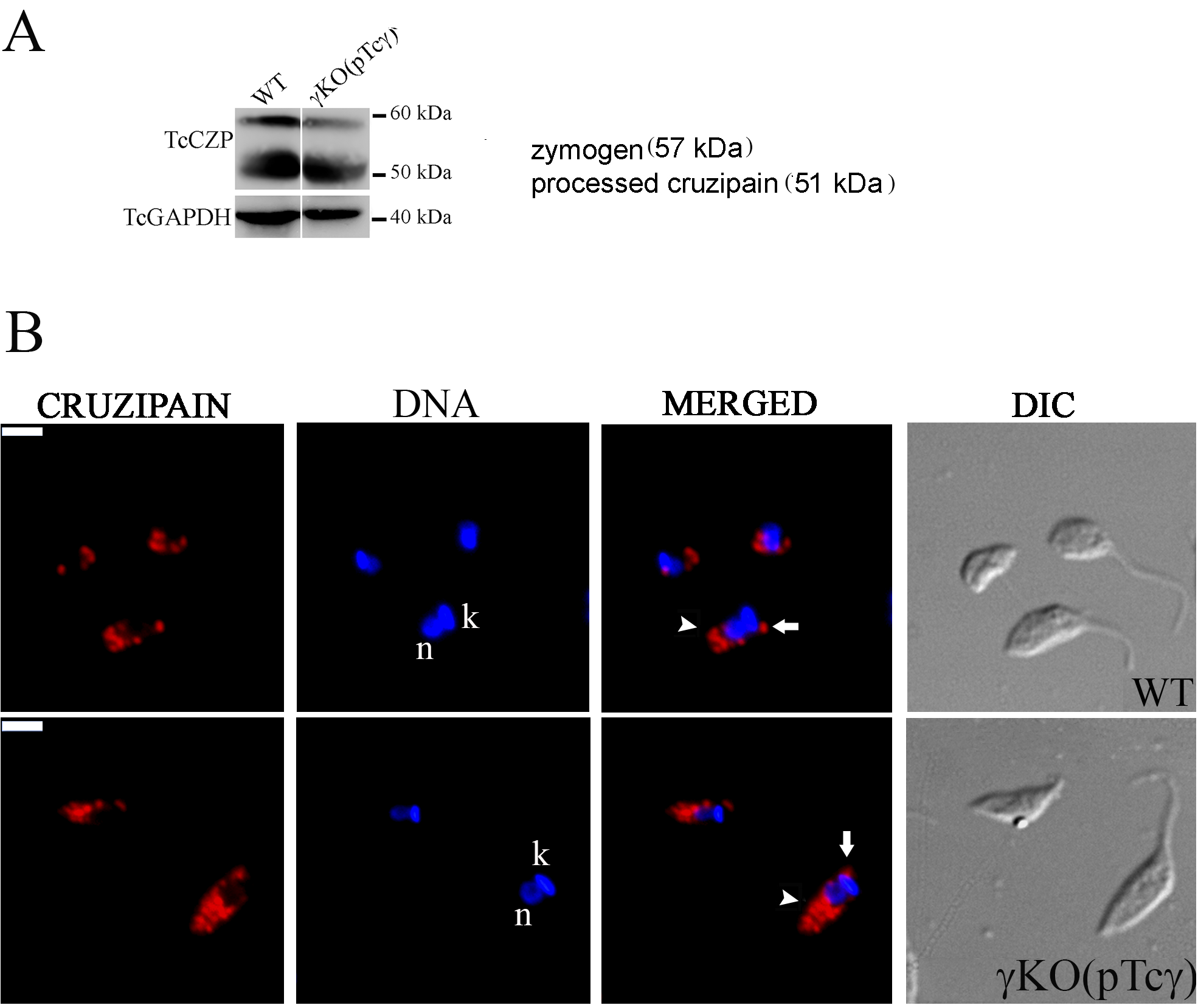

Supplement: S4 Fig — A) Whole cell lysates from wild-type (WT) and the complemented γKO(pTcγ) epimastigotes were separated by SDS-PAGE, transferred to nitrocellulose membranes and labeled with an anti-cruzipain (TcCZP) antiserum. In γKO(pTcγ) epimastigotes, the processing of cruzipain was restored as observed by the presence of a band of 51 kDa corresponding to the mature form of the enzyme. Labeling for the T. cruzi GAPDH was used as a loading control. B) Wild-type (WT) and γKO(pTcγ) epimastigotes were labeled with an anti-cruzipain antiserum, detected with anti-mouse IgG conjugated to Alexa Fluor 594. Cruzipain localized in the region of the Golgi complex (arrows) and reservosomes (arrowheads) in WT and the complemented strain. Nuclear (n) and kinetoplast (k) DNA were stained with Hoechst 33342. DIC, differential interference contrast microscopy. Scale bar = 5μm (TIF) [file pone.0179615.s004.tif]

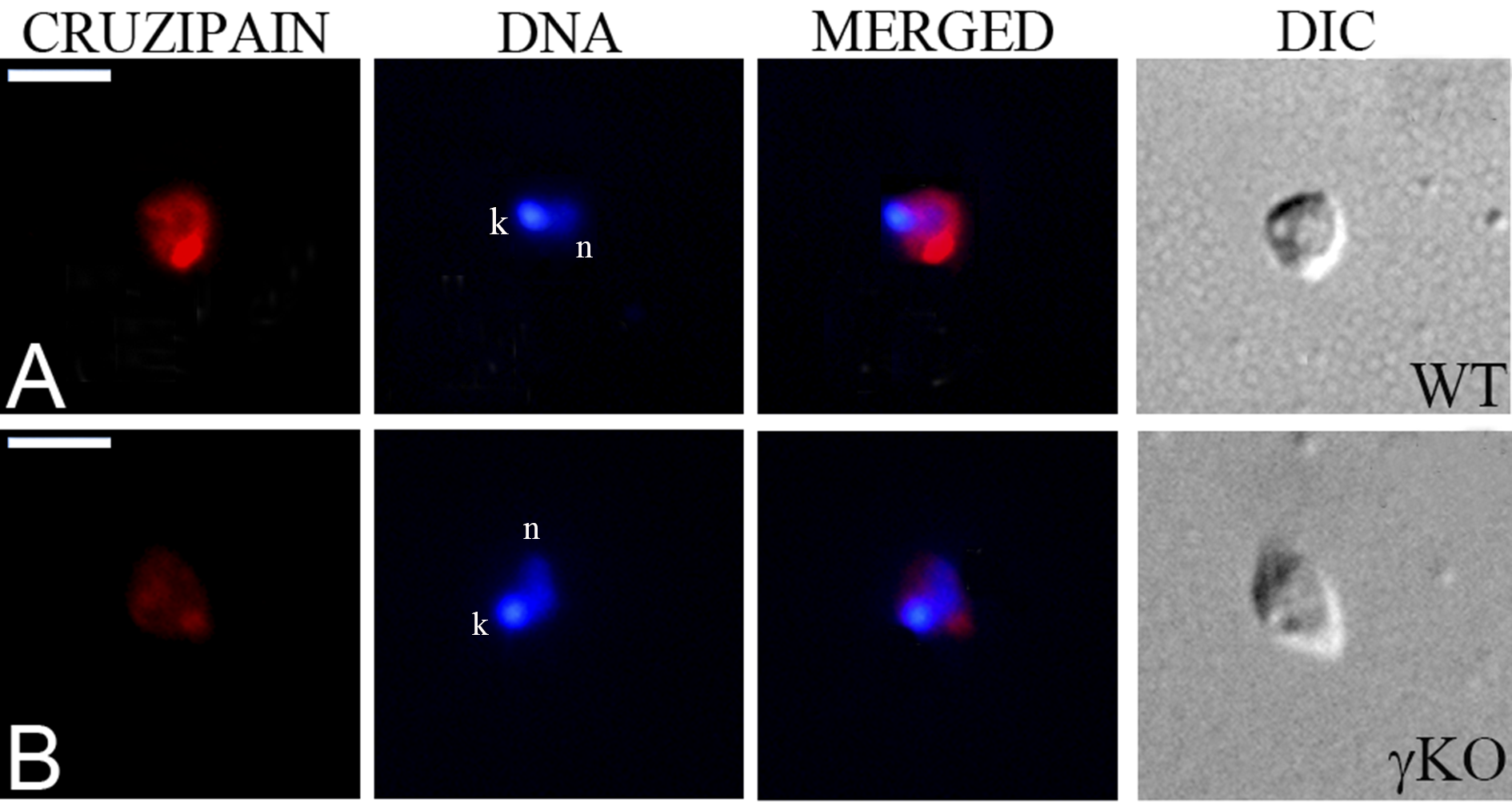

Supplement: S5 Fig — Wild-type (WT) and AP-1 γ null mutant (γKO) amastigote (not permeabilized) were labelled with anti-cruzipain antiserum and, detected with anti-mouse IgG conjugated to Alexa Fluor 594. A strong labelling was localized in the WT surface (A), whereas the γKO amastigote surface was faintly labelled (B). Nuclear (n) and kinetoplast (k) DNA were stained with Hoechst 33342. DIC, differential interference contrast microscopy. Scale bar = 5μm (TIF) [file pone.0179615.s005.tif]

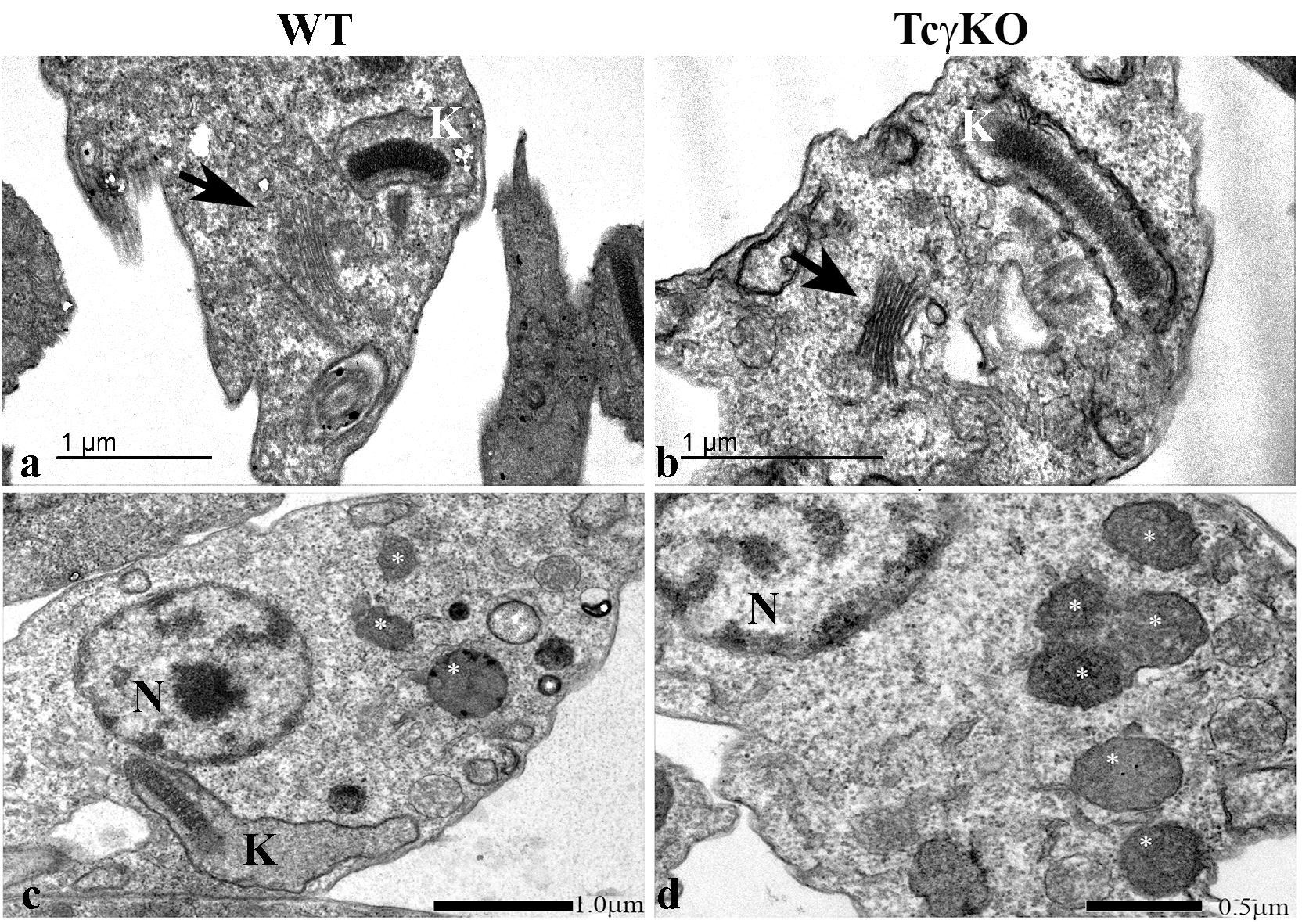

Supplement: S6 Fig — The Golgi complex (arrow) is observed at the anterior region of both WT (a) and TcγKO (b) parasites. No noticeable morphological alteration is observed, except that the Golgi cisternae are more contrasted and easily detected in TcγKO parasites. Reservosomes (*) with electron-dense matrix are found at the cell posterior region in both WT (c) and TcγKO (d) parasites, with no remarkable difference in size, shape and density. N = nucleus, K = kinetoplast. (TIF) [file pone.0179615.s006.tif]
